# Earlier Intervention with Deep Brain Stimulation for Parkinson's Disease

**DOI:** 10.1155/2017/9358153

**Published:** 2017-08-16

**Authors:** Gerson Suarez-Cedeno, Jessika Suescun, Mya C. Schiess

**Affiliations:** Department of Neurology, The University of Texas McGovern Medical School at Houston, Houston, TX, USA

## Abstract

Neuromodulation of subcortical areas of the brain as therapy to reduce Parkinsonian motor symptoms was developed in the mid-twentieth century and went through many technical and scientific advances that established specific targets and stimulation parameters. Deep Brain Stimulation (DBS) was approved by the FDA in 2002 as neuromodulation therapy for advanced Parkinson's disease, prompting several randomized controlled trials that confirmed its safety and effectiveness. The implantation of tens of thousands of patients in North America and Europe ignited research into its potential role in early disease stages and the therapeutic benefit of DBS compared to best medical therapy. In 2013 the EARLY-STIM trial provided Class I evidence for the use of DBS earlier in Parkinson's disease. This finding led to the most recent FDA approval in patients with at least 4 years of disease duration and 4 months of motor complications as an adjunct therapy for patients not adequately controlled with medications. This following review highlights the historical development and advances made overtime in DBS implantation, the current application, and the challenges that come with it.

## 1. Introduction

Idiopathic Parkinson's disease (PD) is the second most prevalent neurodegenerative disorder in the western world. Dopaminergic neuronal loss begins as early as 10 years before motor symptoms appear. Diagnosis is still clinical and relies on the United Kingdom Brain Bank Criteria [1]. Currently there is no therapy to stop disease progression and management is directed primarily at motor symptoms relief. PD has a substantial economic impact on the healthcare system with an estimated cost of drug treatment calculated to be between $1,000 and 6,000 per year and annual healthcare cost between $2,000 and 20,000 per year [2, 3]. A multitude of dopamine enhancing agents are available as therapeutic options and usually employed as the first line of treatment. Although they are very effective in early disease stages there is an increasing awareness of refractory symptoms and well described motor complications related to chronic therapy [4]. These aspects have helped to establish a window of optimal therapeutic benefit for pharmacological approaches. As a result, neuromodulation by DBS arose as an adjunctive therapy for the management of dopamine-responsive patients with advanced disease. Initial use of DBS in advanced disease was heralded as a safe, cost-effective, and adjustable procedure that can be programmed to maximize motor benefits while minimizing side effects [5]. In the past few years the concept of earlier DBS therapy emerged as a therapeutic tool to prevent the development of motor complications and prolong quality of life for PD patients.

## 2. Historical Review

Before the discovery of dopaminergic agents, ablative surgical intervention was the main treatment for the motor symptoms of PD. The origins of the surgical interventions for movement disorders date back to the early twentieth century when the basal ganglia was considered a potential target for surgical intervention. Dr. E. Jefferson Browder at the State University of New York described improvement of Parkinsonian symptoms with caudate nucleus extirpation; and almost two decades later in 1947 neuroscientist Ernest A. Speigel and neurosurgeon Henry T. Wycis at Temple University developed the first stereotaxic frame for humans. In parallel, the Neurophysiologist Jose Delgado at Yale University performed several experiments of deep electrical stimulation in animals and humans for behavioral control.

In 1953 Dr. Cooper made an accidental ligation of the anterior choroidal artery that resulted in a reduction of the contralateral tremor and rigidity in a PD patient [6]. He then proposed that this was due to an infarction of the globus pallidus interna (GPi), and as a consequence pallidotomies became a surgical treatment for PD [7]. Later, other structural lesions were studied leading to the identification of specific thalamic nuclei as a second anatomical target for therapy [8].

The next decade was notable for an expansion of ablative surgery [9] as a reflection of stereotaxic refinement and surgical procedures focused on thalamotomies and pallidotomies. In 1961 W. Watson Alberts at the Institute for the Study of Human Neurophysiology at Mount Zion Hospital studied stimulation thresholds in various parts of the globus pallidus interna and the ventrolateral thalamus. This was followed by a breakthrough discovery by neuroscientist Albe-fessard at Pierre and Marie Curie University who reported that ventralis intermedius (VIM) stimulation between 100 and 200 Hz suppressed tremor in Parkinson's patients.

The first description of chronic thalamic stimulation was made in 1965 by Carl Wilhelm Sem-Jacobsen, a Norwegian neurophysiologist and psychiatrist. However, the introduction of levodopa in 1967 by Cotzias et al. [10] temporarily ended the era of ablative surgery and neuromodulation; dopaminergic agents became the preferred treatment for PD. Dopaminergic therapy revolutionized PD treatment, but over time the limitations and side effects of taking levodopa for more than 5 years emerged. Once the limitations of motor fluctuations and dyskinesia were recognized as a consequence of long-term and high dosage levodopa therapy, there was a renewed interest in surgical therapies.

The idea of using chronic subcortical stimulation as a permanent therapy was developed in the 1970s by Dr. Natalia Petrovna Bechtereva at the Institute of Experimental Medicine and the Academy of Medical Sciences in Leningrad. Dr. Petrovna implanted electrodes into the ventrolateral and centromedian thalamic nuclei and administered intermittent high frequency pulses over several sessions. Unfortunately, since most of her articles were written in Russian and not further translated, her work was not widely disseminated.

The DBS golden era for PD was introduced to neurologists and neuroscientists by work from Dr. Benabid and his colleagues in 1987 at the Grenoble University. Their original paper highlighted the use of the traditional approach of VIM thalamotomy combined with chronic stimulation of the contralateral VIM, resulting in similar suppression of tremor in both affected sides. Afterwards, high frequency stimulation was used on 26 PD patients demonstrating improvement in tremor and rigidity, while dopaminergic medication dosage was reduced by 30% in 10 of these patients [11]. The same group eventually proved subthalamic nuclei stimulation (STN) to be not only a superior target but also the preferred intervention compared to pallidotomy [12] and thalamotomy for PD [13]. Thereafter, in 1994, the neurosurgeon Jean Siegfried at the Klinik Im Park in Zurich reported improvement of multiple symptoms of PD by stimulation of the globus pallidus interna (GPi) [14].

**Figure 1:**
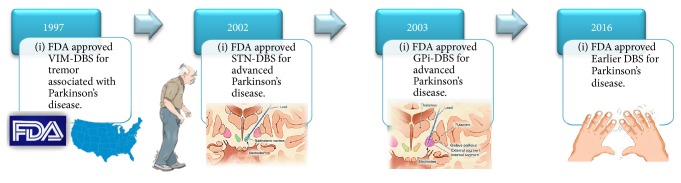
DBS FDA approval timeline.

In 1997, the FDA approved for the first time the use of DBS as therapy in movement disorders, establishing the practice of VIM-DBS to treat essential tremor and tremor associated with Parkinson's disease (Figure 1; DBS FDA approval timeline). The first clinical trials of DBS for PD were done in 1998 by the Grenoble group. They demonstrated sustained improvement of motor fluctuations, dyskinesia, and a decrease of medication dose requirement in patients with PD and bilateral STN-DBS [15]. Simultaneously, Anthony Lang's group at the University of Toronto reached similar conclusions after completing a double-blind study [16]. Okun et al. at the University of Florida reported a retrospective review showing greater motor improvement with STN-DBS compared with GPi-DBS [17].

Three years later a large prospective double-blind study comparing STN versus GPi for PD showed a greater motor benefit from STN-DBS [18]. Collectively, these findings established the basis for how we use DBS therapy today. The level of evidence prompted eventual support by the FDA for STN-DBS in PD in 2002 [19]. Thereafter, the first long-term follow-up study of STN-DBS in PD showed sustained improvement in motor symptoms and activities of daily living [20]. Since then, tens of thousands of patients have undergone DBS implantation [21], and numerous case reports and randomized controlled trials (RCT) have confirmed the long-term efficacy of STN and GPi targeting for the treatment of PD symptoms [17, 22–27]. The current practice parameter guidelines for DBS in PD published by the American Academy of Neurology in 2006 suggest the use of STN-DBS for PD, graded as level C evidence for improving motor function and reducing motor fluctuations, dyskinesia, and antiparkinsonian medication usage [23].

## 3. DBS in the Contemporary Era

Several theories have been proposed to explain the neuroprotective effect of DBS in PD. Despite the vast surgical experience with DBS, its mechanism of action and neuroprotective effects are still poorly understood [28]. DBS has electrical, chemical, and neural network effects. Computational studies have shown a possible simultaneous cell body inhibition with axonal excitation [29]; this decoupling phenomenon resulted in a network activity modification, influencing multiple thalamocortical circuits. The electrical stimulation disrupts pathological basal ganglia activity by changing firing rate [30] and increasing blood flow to the midbrain [31]. At the same time DBS triggers astrocytes to release calcium and neurotransmitters (adenosine and glutamate) and also stimulates neurogenesis [32, 33].

Class III evidence supports DBS therapy as beneficial for nonmotor symptoms such as improving sleep, mood/cognition, pain, and urinary and gastrointestinal symptoms [34–37]. This can be partially explained by increased mobility after surgery and overall improvement in quality of life, in addition to decreased medication needs. The combination of these effects may explain the associated reduction in anxiety and impulse control disorder [38]. However, there is a widely described detrimental effect on phonemic and semantic verbal fluency after the procedure [26].

The paradigm shift for DBS intervention came from two redefining concepts: (1) DBS in addition to best medical treatment (BMT) is more effective than BMT alone and (2) an earlier intervention could preserve functional capacity. Randomized controlled trials (RCT) have shown that DBS plus BMT can be superior to BMT alone, not only for improving motor function during the “off” state measured by UPDRS-III (motor subscale), but also by increasing quality of life (PDQ-39 self-reporting survey), maintaining activities of daily living (ADL), decreasing levodopa requirements, and expanding time spent in the “on” state without troublesome dyskinesia.  Table 1 summarizes the main studies that proved this concept. With the exception of Charles et al. [39], all studies showed significant improvement in DBS patients when compared with BMT alone, ranging from 41% to 71% in the UPDRS-III. The Charles et al. study was designed as a safety study and was not powered to generate efficacy conclusions.

Given the robust response to DBS in PD and the quest to maintain quality of life, multiple studies have addressed the issue of functional capacity and symptoms reduction with the long-term use of DBS. Nonrandomized studies have shown sustained reduction of motor symptoms and levodopa induced dyskinesia after a five-year follow-up [20], motor scores improvement remained present after eight years [45], and medication reduction was still present at ten years after implantation. These studies were limited due to their nonrandomized design. In 2011, Parent et al. published a retrospective study with a subgroup analysis divided by age and disease duration, showing that there was an improvement in rigidity after a one-year follow-up in patients with disease onset less than 10 years versus. longer than 10 years. Similar results were seen in other prospective studies [46].

Thereafter, studies by Schuepbach et al. [44] and Charles et al. [39] explored the innovative concept of off-label early stage DBS. The pioneer study was done by a group from Vanderbilt University that published a pilot case of their early intervention in 2011 [47]. 30 patients between the ages of 35 and 75, Hoehn and Yahr stage II, and dopamine response for more than 6 months but less than 4 years were randomized to receive BMT or DBS plus BMT. The primary endpoints were the time to reach a 4-point change from baseline scores in the UPDRS-III off therapy and the change on levodopa equivalent dose from baseline to 24 months. Final results were published in 2014 [39]: the mean motor scores were not significantly different for on or off therapy and the DBS group required less medication than the BMT group at all time points with a maximal difference seen at 18 months. Two serious adverse effects occurred in two subjects, one subject had a perioperative basal ganglia infarct, and another had a traumatic scalp infection requiring removal of the device. A posterior post hoc analysis was conducted in 2015 [48] including all subjects from the pilot and a subset of subjects taking PD medications 1–4 years at enrollment which showed that DBS plus optimal drug therapy subjects experienced a 50–80% reduction in the relative risk of worsening after two years. Total UPDRS, complication of therapy, and PDQ-39 scores significantly worsened in the BMT group (*p* < 0.003); finally the DBS + BMT group significantly improved in the motor score (UPDRS-III) compared to the BMT (*p* = 0.02). Currently the group is preparing to launch a phase III clinical trial on early stage PD STN-DBS.

In 2012, a German-French group published a paper theorizing three phases of PD progression [49]. The first phase, the honeymoon period, is when the disease is well controlled with medications. The second phase, the intermediate phase, is when patients develop motor complications such as “on/off” fluctuations as a result of chronic dopaminergic therapy; this phase is variable in duration and is determined by individual biological/physiological factors. The third one is the levodopa resistant phase, when physicians struggle to find a trade-off between maximizing motor symptoms control and minimizing the presence of motor complications as a side effect.

The concept of PD phases prompted the initial hypothesis that the use of DBS as an adjunctive therapy in the early stage second-phase disease could maintain quality of life and social adjustment in PD patients, leading to the EARLY-STIM trial [44], an early interventional study in PD. Patients included in this trial were 60 years of age or younger and had onset of PD for 4 years or more, but less than 3 years of motor complications. The initial sample size included 251 patients, who were then randomized to either receive BMT or STN-DBS. The authors of the EARLY-STIM study [44] chose quality of life as their primary outcome measured by the 39 items of Parkinson's disease questionnaire for quality of life (PDQ-39), mainly because it evaluates the influence on quality of life by both motor and nonmotor symptoms of PD. After two years of follow-up the final results were published in the New England Journal of Medicine in 2013: a total of 226 of the 251 patients recruited were analyzed; the others were excluded due to deviation from the protocol or adverse events. Results showed that the PDQ-39 score improved by 26% in the neurostimulation plus BMT group but worsened in the BMT group. UPDRS-III scores improved by 53% in the neurostimulation group versus 4% in the BMT group. Medication dose was reduced 39% in the neurostimulation group but increased 21% in the BMT group. No significant cognitive changes were found between groups. Importantly, depression was more frequent in the neurostimulation group. In addition the study showed decreased progression of motor complications in a selected population between ages 19 and 60 with less than 4 years of disease duration as well as no more than 3 years of motor complications. Summarily, this pivotal study demonstrated additional Class I evidence of sustained motor and quality of life improvement after two years of DBS compared to BMT alone.

These two studies are the backbone of earlier intervention in PD; furthermore there were two Japanese prospective publications that reported significant improvement in ADLs and UPDRS-III with early STN-DBS implantation after 3- and 6-month follow-up [50, 51].

Moreover a base-case analysis showed that the incremental cost-utility ratio for STN-DBS versus BMT was 22.700 euros per quality adjusted life year gained, showing that STN-DBS at earlier stages of the disease is cost-effective in patients below the age of 61 [52]. Likewise a decision analysis model of early versus. delayed bilateral DBS implantation showed that early intervention results in superior cost-effectiveness due to a greater quality adjusted life expectancy by reduction in pharmaceutical cost, therapy, and specialist consultations [53]. Similarly, DBS offered earlier provided substantial long-term reduction in medication cost by maintaining a simplified, low dose medication regimen [54].

The above findings led to the recent FDA approval of DBS in PD levodopa-responsive patients with at least 4 years of disease duration and 4 months of motor complications not adequately controlled with medications [55].

The implementation of this new criterion of early intervention based on the EARLY-STIM criteria requires a complete evaluation of the limitations of this study and has been the subject of extensive ethical discussion [56, 57]. Despite a strong design, the inclusion criteria excluded patients older than 60 years, an age group that has a high risk of rapid development of motor complications. Therefore, clinical decisions in this age group are limited. In addition, long-term expectations for the procedure effect are difficult to predict due to the short follow-up period of only 2 years. This time frame could be considered insufficient when observing a progressive chronic illness such as PD [58]. Future follow-up data from the EARLY-STIM study will help to answer these concerns.

The lack of a double-blinded design with sham surgery raised the concern for placebo effect in this trial. Some authors state that the placebo effect in PD trials can be as high as 39% [59]. However, this number has been extrapolated from PD trials that do not have a DBS surgery therapy component. Motor and quality of life improvement was sustained for two years and the motor assessment was performed with on/off stimulation and rated blindly by a movement disorder specialist. The two-year follow-up reduces the probability of a placebo effect that would prevail for such a long time given the natural history of disease progression [60].

The rate of progression in PD is variable [61–63] but an important concept in order to determine when to offer DBS. PD progression is influenced by many aspects including but not limited to age at diagnosis, gender, genetics, motor subtype, presence of certain symptoms at diagnosis [64], life style, and treatment. There is evidence from two 5-year follow-up studies suggesting that motor complications derived from therapy remain relatively mild in the early years after their onset in dopamine naïve patients [65, 66]. Angeli et al. [67] found in a retrospective review of patients who underwent DBS that Parkin mutation carriers reached motor complications earlier but had a less prolonged course; likewise glucocerebrosidase mutation carriers reached the threshold for DBS earlier and had more cognitive impairment after the procedure. Deciding when to undergo an elective surgical procedure requires a careful consideration of motor complications, rate of progression, and additional therapeutic options and it should be done on a case-by-case basis including a risk versus benefit evaluation by a multidisciplinary team [68].

The motor and nonmotor benefit obtained in the earlier intervention studies is at least as good as or even better than what RCTs have shown in advanced Parkinson's disease. Earlier DBS extends the possibility of maintaining functional capacity and improving the patient's quality of life earlier in the disease course.

## 4. Earlier DBS Intervention Challenges

Within the movement disorders field, the concept of earlier DBS intervention has been a matter of debate among neurologists and there have been multiple challenges to implement it in the clinical scenario.

### 4.1. Patient Selection

For patients to be considered for early DBS implantation, they require a diagnosis of at least 4 years of disease duration and after 4 months of motor complications, which are not adequately controlled with medication. This 4-year time window has been established to avoid DBS implantation in patients with Parkinson's plus syndromes. This is supported by the literature which shows that most Parkinson's plus syndromes receive correct diagnoses within 4-5 years [69]. In this regard, it is important to keep in mind that diagnostic accuracy performed by MD experts range from 79.6% after the initial assessment to 83.9% after the follow-up [70]. Using the MDS 2015 task force diagnostic criteria for PD, the specificity reaches 90% [71]. This explains why the UK Brain Bank criteria and an on/off trial assessment administered by experienced MD specialist is still the most important outcome predictor for DBS success [72] and avoids implantation of patients with atypical Parkinsonism.

### 4.2. Predictors of Outcomes after DBS

Preoperative indicators for good outcomes in DBS for PD include younger age, short disease duration, robust levodopa-response, few axial motor symptoms, absence of dementia, and stable psychiatric conditions [23]. The EARLY-STIM trial showed motor and quality of life improvement greater than BMT sustained for 2 years.

Patients who will most likely benefit from early DBS intervention according to the EARLY-STIM trial subgroup analysis [73] are patients with baseline poor Hoehn and Yahr scores (stages 4-5) and fluctuating disease (even if only mild) and patients who report poor mobility during a large part of the day.

### 4.3. Adverse Events

The benefit to risk ratio is an important consideration; the use of earlier DBS should be considered in specific patients if the benefits of the surgical therapy are weighed against the procedure risks and the lifelong need for specialized care [73]. DBS complications have been widely reported in advanced Parkinson's patients [40, 74]; these can be categorized as surgery related, hardware related, and stimulation dependent. The most common ones are cerebral hematoma (0–5%), infection (0–15%), skin erosion (1–2.5%), and mental status/behavioral changes (9–18%).

To the best of our knowledge there is no data available regarding a difference in the incidence of these adverse events with an earlier intervention. However, a recent report of the Implantable Systems Performance Registry (ISPR), a prospective, long-term multicenter registry supported by Medtronic® compared adverse events in the overall DBS cohort versus early PD-DBS patients (<7.5 years disease duration) showing no significant differences of the adverse event profile between the Earlier PD Subset and the Overall Cohort [75]. Adverse event rates in the two aforementioned RCT studies were similar to what has been reported in the literature for advanced PD with the exception of a substantial increase in suicidal ideation, attempts, and complete suicides. Evidence from retrospective studies has shown the safety of DBS [76], and RCT of advanced Parkinson disease interventions revealed no elevated suicide behaviors in the 6-month period after DBS surgery [24, 77]. A multicenter retrospective survey of fifty-five movement disorder centers around the world revealed 0.45% suicides and 0.90% suicidal attempts in the following 4 years after STN-DBS [78]. These findings raised the need for psychiatric assessment and close follow-up that may only be successfully performed by an interdisciplinary highly experienced center [79].

### 4.4. Prognosis

Little has been described of the impact of DBS on survival, and it is still a topic of debate that requires further studies. Schupbach et al. published a retrospective study on a historical comparison of 118 operated patients with 39 nonoperated patients from a different population; survival among operated patients was not different compared to 118 nonoperated patients with overlapping ages at PD onset (HR = 1.2; 95% CI = 0.7–2.1) [41]. In addition, Lilleeng et al. compared mortality over time in two matched groups of PD patients with and without DBS and found that survival was similar in the two groups during long-term follow-up (HR = 1.76, CI = 0.91–3.40, *p* = 0.091) [80]. In contrast, Ngoga et al. conducted a controlled trial and concluded that age matched patients that underwent STN-DBS had significantly longer survival and were significantly less likely to be admitted to a residential care home than those managed purely by a medical regimen (survival: *p* = 0.002, HR 0.29 [95% CI: 0.13 to 0.64]) [81].

### 4.5. Neuroprotection

Several animal models have raised the possibility of DBS as a neuroprotective therapy. Multiple studies of STN-DBS on 6-OHDA lesioned rodents showed that rats with DBS had less dopaminergic cell loss compared with controls [82–85]. Another study in a MPTP primate model reported that up to 24% of dopaminergic cells were preserved following STN-DBS compared with controls [86]. On the other hand clinical studies have not been able to prove the same concept; a multicenter international DOPA-PET-study did not show any reduction in the loss of dopaminergic terminals [87] and multiple clinical trials reported an increase in motor symptoms over time with DBS. Nonetheless, the animal studies best represented a moderate stage of disease and not the extreme nigral cell loss seen in advanced PD [88].

## 5. Conclusion

Deep Brain Stimulation for Parkinson's disease was developed based on findings from ablative surgical procedures. Research into its use decreased with the advent of levodopa but resumed in the early 1990s due to frequent motor complications and symptoms refractory to dopaminergic therapy. In 2002, DBS was approved for late stage PD with motor complications. Even more recently in 2016 Class I evidence led to the approval for earlier intervention in patients who were diagnosed for at least 4 years and exhibited at least 4 months of motor complications. Early interventions require the assessment and follow-up of an interdisciplinary and highly experienced team. Due to the recent approval of earlier intervention, we are missing knowledge regarding the patient progression and the long-term outcomes of the early DBS patients. Nonetheless, extensive education of the healthcare community, especially neurologists, is crucial in order to provide the intervention for appropriately selected candidates. Earlier DBS intervention offers the opportunity to impact PD patients' quality of life and functional ability, providing potential significant symptomatic relief over a longer period of time.

## Figures and Tables

**Table 1:** Randomized controlled trials for DBS versus BMT in PD.

Study	Target/number	Mean age (yrs)	Baseline characteristic in the “off” state	Mean disease duration (yrs)	Follow-up (mos)	Outcome/conclusion
Deuschl et al. 2006 [40]	STN + BMT: 78 BMT: 78	STN + BMT: 60.5 BMT: 60.8	*H & Y* STN + BMT: 3.7, BMT: 3.8 *UPDRS-III* SNT + BMT: 48.0, BMT: 46.8	>5	6	(i) UPDRS-III: 41% improvement in DBS versus 0% in the BMT (*p* < 0.001). (ii) PDQ-39: STN resulted in 14 % improvement.

Schüpbach et al. 2007 [41]	Bilateral STN + BMT: 10 BMT: 10	Bilateral STN + BMT: 48.4 BMT: 48.5	*H & Y* <3 *UPDRS-III* Bilateral STN + BMT: 32.7 BMT: 25.3	Bilateral STN + BMT: 7.2 BMT: 6.4	18	(i) UPDRS-III: 69% improvement in DBS versus worsening in BMT (*p* < 0.05). (ii) PDQ-39: 24% improvement in DBS versus 0% in BMT (*p* < 0.05). (iii) Levodopa dose: reduced by 57% in the DBS versus 12% in the BMT (*p* < 0.001).

Weaver et al. 2009 [42]	Bilateral STN/GPi: 121 BMT: 134	Bilateral STN/GPi: 62.4 BMT: 62.3	*H & Y* Bilateral STN/GPi: 3.4, BMT: 3.3 *UPDRS-III* Bilateral STN/GPi: 43 BMT: 43.2	Bilateral STN/GPi: 10.8 BMT: 12.6	6	(i) UPDRS-III: 71% of DBS patients experienced clinically meaningful motor function versus 32% of BMT patients (*p* < 0.001). (ii) PDQ-39: DBS group had significant improvement (*p* < 0.001).

Williams et al. 2010 [43]	Bilateral STN/GPi: 183 BMT: 183	DBS: 59 BMT: 59	*H & Y* DBS: 3.1, BMT: 3.2 *UPDRS-III* DBS: 47.6, BMT: 48.6	DBS: 11.5 BMT: 11.2	12	(i) UPDRS-III: 36% improvement in the DBS group versus 2% in BMT (*p* < 0.0001). (ii) PDQ-39: mean improvement compared with baseline was 5.0 for the DBS group versus 0.3 points in the BMT (*p* = 0.001).

Schuepbach et al. 2013 [44]	SNT + BMT: 124 BMT: 127	SNT + BMT: 52.9 BMT: 52.2	*H & Y* <3 *UPDRS-III* STN + BMT: 33.2 BMT: 33	STN + BMT: 7.3 BMT: 7.7	24	(i) UPDRS-III: 56% improvement in the DBS group versus 4% in BMT (*p* < 0.001). (ii) PDQ-39: 26% improvement in the DBS group versus no improvement in the BMT group (*p* = 0.002). (iii) Levodopa induced motor complications: 61% improvement in the DBS group versus no improvement in the BMT group (*p* < 0.001).

Charles et al. 2014 [39]	STN + BMT: 15 BMT: 15	STN + BMT: 60 BMT: 60	*H & Y* STN + BMT: 2, BMT: 2 *UPDRS-III* STN + BMT: 25.3 BMT: 25.6	STN + BMT: 2.2 BMT: 2.1	24	(i) UPDRS-III: mean scores were not significantly different on or off therapy. (ii) Medication requirements in the DBS + BMT group were lower at all time points.

DBS: Deep Brain Stimulation; SNT: subthalamic nucleus; GPi: globus pallidus interna; BMT: best medical therapy.
